# Gene Expression Changes with Minor Effects on the Population Average Have Major Effects on the Occurrence of Cells with Extreme Protein Concentrations

**DOI:** 10.1128/mSphere.00575-18

**Published:** 2019-01-30

**Authors:** Mikkel Skjoldan Svenningsen, Szabolcs Semsey, Namiko Mitarai

**Affiliations:** a The Niels Bohr Institute, University of Copenhagen, Copenhagen, Denmark; b Department of Biology, University of Copenhagen, Copenhagen, Denmark; University of Iowa

**Keywords:** distribution, noise, rare events

## Abstract

The cell-to-cell heterogeneity in a bacterial population provides a rich response to environmental changes and robust survival of an isogenic population. Especially, the rare, extreme phenotypes can be important for survival under transient lethal conditions. We analyze the probability of having an extremely high or low protein level in a stochastic model of gene expression. The fraction of rare state cells defined as the cells in the tails of distributions is found to be highly sensitive to small changes of the mean protein level. The result highlights the importance of relatively weak changes to the mean for the occurrence of rare phenotypes.

## Abstract (precis)

The cell-to-cell heterogeneity in a bacterial population provides a rich response to environmental changes and robust survival of an isogenic population. Especially, the rare, extreme phenotypes can be important for survival under transient lethal conditions.

## OPINION/HYPOTHESIS

A large population of bacteria enables us to detect rarely occurring phenotypes experimentally. Examples of rare phenotypes include the well-known bacterial persistence that occurs in as rarely as 1 in 10^6^ cells and allows survival at otherwise lethal doses of antibiotics (1) or sporulation in a subpopulation of Bacillus subtilis under strong starvation stress (2). More broadly, a small subpopulation of cells containing much less or much more of a given protein than the population average can have a significant consequence in the overall population’s growth and survival, by allowing them to avoid phage attack (3, 4), to survive in the presence of antimicrobials (5–7), or to respond faster to nutrient shifts (8–10). Such heterogeneity of phenotypes can occur due to inevitable noise in gene expression (11), or it can evolve as a regulated bet-hedging strategy: i.e., investing a subpopulation into various phenotypes that are beneficial for different environments in order to survive a sudden environmental change (12, 13).

It has been extensively studied how cells’ behaviors depend on the noise in gene expression (14, 15). Typically the noise is characterized by using the ratio between the standard deviation and the mean (11, 16) or the Fano factor (15, 16), which characterize the “typical” deviation of the protein concentrations from the mean concentrations. There have also been attempts to characterize the full distribution based on mathematical models (17–19) and experiments (16, 19).

For a typical protein distribution (16, 19), a seemingly small change in the protein distribution shape can have a paramount impact on the probability of rare concentrations. Because this is associated with the tails of the protein distributions, we refer to this as the “tail effect.” However, despite this observation and the biological importance of rare phenotypes, the dependence of the extreme fractions on the various parameters in gene expression has received little attention.

Here, we revisit the protein distribution in an isogenic population in a simple mathematical model, with a focus on the extreme events that occur with small probability. We analyze the fraction of “rare-state cells,” which have a rare number of a given protein compared to the rest of the population. In a bacterial culture, the cell count can easily exceed 10^9^ cells/ml, meaning that the rare events that occur with a probability as low as 1 in 10^9^ can still be observed. Although they are rare and often transient, these events can play a crucial role if the rare state of interest provides advantages in survival. We demonstrate the strong sensitivity of the fraction of the rare-state cells to the various parameters that result in changes in the mean.

## MODELING

### The rare-state cells in the steady state.

We consider a simple model of stochastic gene expression (19, 20):

(1) $$\text{DNA}\overset{\alpha }{\to }\underset{{}_{\emptyset }^{↓\gamma_{m}}}{\text{mRNA}}\overset{\beta }{\to }\underset{{}_{\emptyset }^{↓\gamma_{p}}}{\text{protein}}$$

Here, mRNAs are transcribed at rate α and degraded at rate γ*_m_*, each mRNA is translated to produce proteins at rate β, and the proteins are degraded or diluted at rate γ*_p_*. We use parameters typical for the bacterium Escherichia coli (20), where mRNA lifetime is much shorter than the protein dilution time: hence multiple proteins are produced as a burst after one mRNA is transcribed. Because this “burstiness” strongly affects the protein number fluctuation (16, 17), the transcription and the translation contribute differently to the distribution. For that reason, changes in the protein distribution tails are analyzed for both changes in the transcription rate, α, and the translation rate, β. To quantify the dependence of the distribution tail on the model parameters, we numerically integrate the corresponding master equation (see Methods) to obtain the distributions.

**FIG 1:**
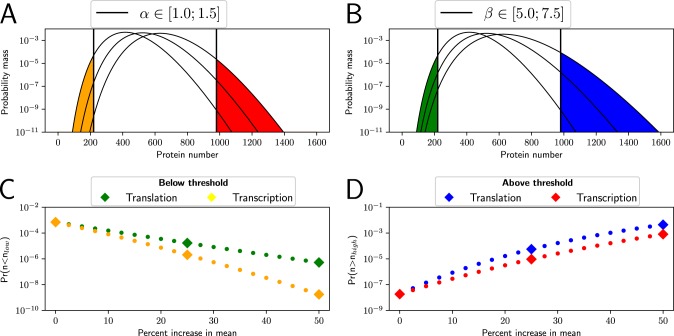
The occurrence of rare states is strongly dependent on the mean protein number. We assume the E. coli volume is ∼1 μm^3^; hence, one protein per cell corresponds to the concentration of 1 nM. Unless otherwise noted, parameters are fixed to α = 1/min, *γ_m_* = 0.35/min, γ*_p_* = 1/30/min, and β = 5/min. (A) Protein distribution with 3 different values of the transcription rate (α = 1/min, 1.25/min, and 1.5/min). A high threshold and low threshold are indicated by vertical lines and are the same for both panels A and B. (B) Protein distribution with 3 different values of the translation rate (β = 5/min, 6.25/min, and 7.5/min). The three different means are the same in panels A and B. (C) The probability to be below a threshold, upon changing the transcription rate, α (yellow), or the translation rate, β (green), to have a different protein mean. The effect of changes in transcription has a stronger impact than changes in translation. (D) The probability to be above the threshold, upon changing the transcription rate, α (red), or the translation rate, β (blue), to have a different protein mean. The three distributions depicted in panels A and B are represented by diamonds in panels C and D.

The example protein number distributions in the steady state are shown in Fig. 1. In Fig. 1A, we show the three distributions, where the protein mean number is altered by changing the transcription rate, α, without changing other parameters. For comparison, in Fig. 1B, we show the distribution of the protein number with the same means, but by changing the translation rate, β.

The different outcomes with regard to the probability of tail events are due to their different degrees of burstiness. Increasing the average by more frequent transcription (higher α) averages out the noise from bursty production, while the increase of β results in more protein production per mRNA, increasing the burstiness and hence increasing the frequency of the rare tail events.

To quantify the rare states, we draw two thresholds, *n*_low_ and *n*_high_. We see that the fractions of the population beyond the thresholds change drastically upon changing the mean moderately. This sensitivity is depicted quantitatively in Fig. 1C by plotting the probability to have protein below *n*_low_ (or above *n*_high_ [Fig. 1D]) as a function of the mean number of proteins.

For all the cases, we observe change of several orders of magnitude in the extreme population fraction upon changing the mean by only up to 50%. The difference in the α and β dependence is consistent with the fact that an increase of β results in a stronger increase of noise than an increase of α: The rare states below a threshold with increasing β do not decrease as steep as the case with increasing α, even though the population mean is going away from the threshold *n*_low_, because the distribution is widening more with β. Similarly, the widening distribution with increasing β increases the probability to be above *n*_high_, steeper than the case with increasing α.

The sensitivity of the tails to the parameter change can easily also be confirmed in regulated systems. We summarize the effect in autorepressed systems in the supplemental material.

### Residence time in a rare state.

The duration a cell stays in the extreme state is often also important. A good example is the survival of a phage attack by bacteria not expressing the phage receptors (3); if a cell can stay in a zero-phage-receptor state for one cell generation time under exposure to phage, that cell will be able to give rise to two uninfected new cells. This motivates us to examine the probability to have zero proteins for a time scale of a cell generation.

**FIG 2:**
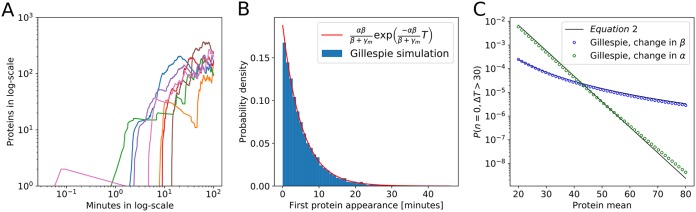
Probability to have zero proteins for the duration of a cell generation time. Unless otherwise noted, parameters are set to be α = 0.2/min, β = 10/min, γ*_m_* = 0.35/min, and γ*_p_* = (1/30)/min. (A) Example trajectories of protein numbers per cell over time when the cell had initially zero mRNA and proteins. (B) Distribution of time intervals before the first protein production. The distribution resembles the exponential distribution expected for the waiting for the first mRNA to appear. (C) Probability of a cell having zero proteins for more than 30 min when the mean number of proteins harbored by cells in the population is altered by varying the transcription rate, α (green), or the translation rate, β (blue). Gillespie simulations (symbols) agree well with the analytical expression in equation 2, with *τ_c_* = 30 min.

Figure 2A depicts several trajectories of protein number in a single cell over time that has zero protein and zero mRNA at time zero obtained by the Gillespie method (21). With the present parameter set where the mean protein production per mRNA $\frac{\beta }{\gamma_{m}}$ is significantly larger than 1, most cells will produce a protein soon after the first mRNA is produced. This means that the first protein production is dominated by a Poisson process with the rate $\frac{\alpha \beta }{\gamma_{m}+\beta }$, which corresponds to the rate of mRNA production, α, with a correction for the probability to produce a protein before degrading the mRNA, $\frac{\beta }{\gamma_{m}+\beta }$. Thus, the probability to stay in the zero-protein state decays exponentially with *t* as $e^{\frac{-\alpha \beta }{\gamma_{m}+\beta }t}$ (Fig. 2B).

Figure 2C shows the probability to have zero proteins for a duration of Δ*T* longer than *τ_c_* = 30 min, *P*(*n*=0, Δ*T* > τ_*c*_), in the steady state obtained by Gillespie simulation. Only cells with zero proteins at time zero are considered, and each of these cells that resides in the zero-protein state for 30 min or more is included. We see that when the protein mean is reduced from 80 to 20, such a population fraction increases several orders of magnitude. We again see the exponential dependence on α, while there is weaker dependence on β. Qualitatively this is natural, because we already know that the steady-state probability to be below a threshold value depends exponentially on α as in Fig. 1C, and the duration of time that a bacterium has zero proteins depends exponentially on α. Using the steady-state probability to have zero proteins in reference 18, the approximate form of the distribution (see the supplemental material for derivation) is

(2) $$P(n=0,\Delta T>\tau_{c})={\left(\frac{1}{1+\beta \gamma_{m}}\right)}^{\frac{\alpha }{\gamma_{p}}}\exp\left(\frac{-\alpha \beta }{\gamma_{m}+\beta }t\right)$$

For β >> γ*_m_*, expression 2 has a weak dependence on β and a strong dependence on α, confirming a much stronger effect from changes in the transcription rate compared to changes in the translation rate. Equation 2 is compared to the Gillespie simulation in Fig. 2C.

## CONCLUSIONS

We have demonstrated that the fraction of cells in a population that has an extremely high or low protein level is remarkably sensitive to changes in the protein production parameters that do not affect the mean protein level of the population much. When the mean protein level is changed by a transcriptional or translational regulation by 50%, the fraction of the cells in the rare state can easily change by several orders of magnitude. This insight underscores a need to consider the potentially large effects on the tails of a distribution of cells, when a weak regulatory effect on the population average is observed, if the cells in the tail of the distribution have an interesting phenotype.

For example, in transcriptome-wide studies that analyze changes in gene expression in response to an external stimulus, the threshold for detection of the expression change is often chosen to be relatively high (e.g., 2-fold) to avoid the experimental noise. However, the present analysis shows that even a 10% change in the mean protein level can be enough to cause an order of magnitude change in the tail population fraction. This gap between the response in the mean and the tail may make it challenging to reveal the regulatory links responsible for the rare phenotypes.

An interesting example of the tail effect is the observation that bacterial quorum-sensing signals repress the mean λ phage receptor level by only 40%, but the fraction of cells in the quorum-sensing-induced bacterial population that survived incubation with λ_vir_ increased by more than 3-fold (22). Although not as pronounced as the change of several orders of magnitude reported here, it is likely that the stronger effect on the survival than the mean is partly due to the distribution tail effect.

Of course, we should be cautious about the relationship between the rare protein concentrations in a cell and a rare phenotype.

For example, the bacterial persistence against antibiotics can be a global effect on the cellular physiology, and the probability of a single protein taking an extreme value may be less relevant. At the same time, the extremely low fraction of persisters indicates that they belong to a tail of some kind of distribution, and in general, the fraction of the extreme in a distribution tends to be sensitive to the parameter change. The growth phase has an effect of several orders of magnitude on the persister frequency (23), which can be partly due to the tail effect.

Another important point to consider when applying the tail effect concept to phenotypes is how the threshold between a main population and a rare phenotype is determined. In this study, we chose a hard threshold between the bulk and a rare protein concentration, where plus/minus a single protein at the threshold makes the difference. The tail effect is a general feature of most probability distributions with a hard threshold, so it is not surprising to find it in protein distributions. Even though the hard threshold is highly idealized, we point out here that approximately hard thresholds could possibly be observed in biological systems. The evasion of a phage attack is a good candidate, where the lack of a receptor protein should protect the cell from a lethal infection. Other candidates are regulations by heterocomplex formation with strong binding, as seen, for example, in type II toxin-antitoxin systems (24) and regulation of translation by small RNAs (25), where one component exceeding another in numbers changes the phenotype, resulting in ultrasensitivity.

Obviously, softer thresholds can obstruct the tail effect. In the supplement, we show this for thresholds defined by a Hill function associated with complex formation, an exponential function associated with a constant probability per protein for phenotype change, or by having an even softer log-scale threshold where a difference of an order of magnitude in the protein level is required for the phenotype change. Naturally, the softer the threshold, the weaker the effect, and the sensitivity of the tail to the mean disappears for a log-scale threshold. The notion of a tail effect can thus aid in understanding the biological function of various types of thresholds, where hard thresholds lead to ultrasensitive dependence on the mean protein distribution, whereas rare phenotypes given by soft thresholds are less sensitive to small changes.

## METHODS

The master equation for the probability *P*(*m,n;t*) to have *m* mRNAs and *n* proteins at time *t* is given by

$$\frac{\partial }{\partial t}P(m,n;t)=\alpha [P(m-1,n;t)-P(m,n;t)]+\gamma_{m}[(m+1)P(m+1,n;t)-mP(m,n;t)]+\beta [P(m,n-1;t)-P(m,n;t)]+\gamma_{p}[(n+1)P(m,n+1;t)-nP(m,n;t)]$$

The distributions were obtained by numerically integrating the master equation over time. Maximum *m* and *n* in computation were chosen to be large enough so that the probability to take the maximum values stayed zero. The probability distribution for protein number is given by *P*(*n;t*)=Σ*P*(*m,n;t*).

All solutions are found with the initial condition that the cell contains both zero mRNAs and zero proteins of the gene in question: i.e., all solutions are found for the initial condition that *P*(*m,n;t*)=δ_*m*,0_δ_*n*,0_, where δ*_i_*_,_*_j_* is the Kronecker delta function. The steady-state value is found at 500 min or more.

10.1128/mSphere.00575-18.1 TEXT S1 The derivations for the duration of the zero-protein state and summary of the tail effect in autoregulated systems and the tail effect with soft thresholds. Download Text S1, PDF file, 0.2 MB. Copyright © 2019 Svenningsen et al. 2019 Svenningsen et al. This content is distributed under the terms of the Creative Commons Attribution 4.0 International license.

10.1128/mSphere.00575-18.2 FIG S1 The tail effect in the autoregulated systems. Small changes to the mean, due to changes in the dissociation constant (*K_D_*), give a tail effect. The parameters α = 1/min and β = 5/min were used for these results. Download FIG S1, EPS file, 0.2 MB. Copyright © 2019 Svenningsen et al. 2019 Svenningsen et al. This content is distributed under the terms of the Creative Commons Attribution 4.0 International license.

10.1128/mSphere.00575-18.3 FIG S2 The tail effect in the autoregulated systems with the same mean. (A) Protein distributions with autoregulation all with the same mean. The shape of the distributions is changed with different combinations of the transcription rate, α, and the dissociation constant, *K_D_*. (B) The tail effect for a low threshold (*n* = 0) and high threshold (τ*_h_* = 400). The shape of the protein distribution has a huge effect on the probability for the zero-protein state. The effect is much smaller for the high threshold. The small increase in the probability at very low dissociation constants is presumably due to a higher variance associated with burstiness in the mRNA production. Download FIG S2, EPS file, 0.1 MB. Copyright © 2019 Svenningsen et al. 2019 Svenningsen et al. This content is distributed under the terms of the Creative Commons Attribution 4.0 International license.

10.1128/mSphere.00575-18.4 FIG S3 Variance in protein and mRNA distributions as a function of the dissociation constant. (A) Relation between the dissociation constant and variance for the negatively autoregulated protein distributions. (B) Relation between the dissociation constant and variance for the negatively autoregulated mRNA distributions. (C) Fano factor for the mRNA distributions. Download FIG S3, EPS file, 0.06 MB. Copyright © 2019 Svenningsen et al. 2019 Svenningsen et al. This content is distributed under the terms of the Creative Commons Attribution 4.0 International license.

10.1128/mSphere.00575-18.5 FIG S4 The tail effect in protein distributions following the activation of an autoregulated system. (A) The mean behavior after activation for the various autoregulated systems. (B) The tail effect associated with the transient overshoot in the protein level. The threshold for rare concentrations is set to 450 proteins. (C) The ratio between the maximum probability for the rare concentration and the steady-state probability. For some systems, the probability is transiently elevated more than 1,000-fold. Download FIG S4, EPS file, 0.1 MB. Copyright © 2019 Svenningsen et al. 2019 Svenningsen et al. This content is distributed under the terms of the Creative Commons Attribution 4.0 International license.

10.1128/mSphere.00575-18.6 FIG S5 Hill function as the threshold. Shown is the tail effect with a soft threshold. Here, the soft threshold is modeled as a Hill function of cooperativity 8. Download FIG S5, PDF file, 0.04 MB. Copyright © 2019 Svenningsen et al. 2019 Svenningsen et al. This content is distributed under the terms of the Creative Commons Attribution 4.0 International license.

10.1128/mSphere.00575-18.7 FIG S6 The tail effect modified by the softness of the threshold. Here, the soft threshold is modeled as an exponential function. The change in probability is almost 5 orders of magnitude with a sharp threshold, but decays to approximately zero when the threshold becomes too soft. Download FIG S6, EPS file, 0.1 MB. Copyright © 2019 Svenningsen et al. 2019 Svenningsen et al. This content is distributed under the terms of the Creative Commons Attribution 4.0 International license.
