# Supplementary material for: Gene Expression Changes with Minor Effects on the Population Average Have Major Effects on the Occurrence of Cells with Extreme Protein Concentrations
Source: mSphere. 2019 Jan 30;4(1):e00575-18. doi: 10.1128/mSphere.00575-18 (PMC6354807; doi:10.1128/mSphere.00575-18)
Supplement: TEXT S1 [file mSphere.00575-18-s0001.pdf]

Supplementary material:
Gene expression changes with minor effects on the population average have major effects on the occurrence of cells with extreme protein concentrations

Mikkel S. Svenningsen, Szabolcs Semsey, Namiko Mitarai

0.1 Single cells in time: the zero-protein state

In this section, we consider a cell that do not have the mRNA and the protein of interest at time zero, and calculate the time dependent probablity for the cell to stay in the zero protein state.
A simplified master equation can be constructed for the zero-protein state. The mRNA level follows the same master equation as for the constitutive system presented in the article. But since we are only interested in cells without the protein of interest, and therefore not interested in the actual number of the protein in the cells, we can construct two states for the protein level, the zero and the non-zero state. We consider the probability for the cell to have zero protein and m mRNAs at time t, $P(m,t)$; note that the probability of cells without a protein, $Pr(\text{zero proteins}) = \sum_m P(m,t)$, decrease from one as time goes, since the probability flux leaks to the non-zero protein state. To simplify the model, there is no flow from the non-zero to the zero-state, which is an adequate approximation for distributions with means not too close to zero. The simplified master equation is given in 0.1.

$$\partial_t P(m,t) = \alpha \cdot P(m-1,t) + \gamma_m \cdot (m+1) \cdot P(m+1,t) - (\alpha + \gamma_m \cdot m) \cdot P(m,t) - \underbrace{\beta \cdot m \cdot P(m,t)}_{\text{flow to non-zero state}} \tag{0.1}$$

This equation can be rewritten and solved with the use of the generating function as described in [3] as

$$G(s,t) = \sum_{n=0}^{\infty} s^m P(m,t)$$

Using the master equation 0.1, we obtain a partial differential equation (PDE) for the generating

function as

$$\partial_t G(s,t) = \alpha(s-1)G(s,t) + (-(\gamma_m+\beta)s + \gamma_m)\partial_s G(s,t) \tag{0.2}$$

This can be solved using the method of characteristics, as described by Van Kampen [3]. Using t as the free parameter, the characteristics are:

$$\begin{aligned} d_t s &= (\gamma_m+\beta)s - \gamma_m \\ d_t G &= \alpha(s-1)G \end{aligned} \tag{0.3}$$

The solution to the first equation is given by the sum of the particular and the homogeneus solution, as described in [5]:

$$s(t) = k\exp\left((\gamma_m+\beta)\cdot t\right) + \frac{\gamma_m}{\gamma_m+\beta} \tag{0.4}$$

By inserting 0.4 in equation 0.3, we obtain

$$\begin{aligned} d_t G &= \alpha\left(ke^{(\gamma_m+\beta)t} + \frac{\gamma_m}{\gamma_m+\beta} - 1\right)G \Rightarrow \\ G &= G_0\exp\left[\alpha\left(\frac{k}{\gamma_m+\beta}e^{(\gamma_m+\beta)t} + \frac{-\beta t}{\gamma_m+\beta}\right)\right] \end{aligned} \tag{0.5}$$

By applying the initial condition $G(s,0) = \sum_n s^n P(n,0) = \sum_n s^n \delta_{n,0} = 1$, which corresponds to the assumption that all cells contain zero proteins at $t=0$, to 0.5 we obtain

$$G = \exp\left[\alpha\left(\frac{k}{\gamma_m+\beta}\left(e^{(\gamma_m+\beta)t} - 1\right) + \frac{-\beta t}{\gamma_m+\beta}\right)\right] \tag{0.6}$$

Now k is known from the expression 0.4:

$$k = \exp\left(-(\gamma_m+\beta)t\right)\left(s - \frac{\gamma_m}{\gamma_m+\beta}\right)$$

This is inserted into expression 0.6:

$$G = \exp\left[\underbrace{\alpha\left(\frac{s}{\gamma_m+\beta}\left(1 - e^{-(\gamma_m+\beta)t}\right)\right)}_{s\cdot f_1(t)}\right]\underbrace{\exp\left[\alpha\left(\frac{\gamma_m}{(\gamma_m+\beta)^2}\left(1 - e^{-(\gamma_m+\beta)t}\right) + \frac{-\beta t}{\gamma_m+\beta}\right)\right]}_{f_2(t)}$$

The generating function is expanded in the variable s:

$$G = f_2(t) \sum_{m=0}^{\infty} \frac{(sf_1(t))^m}{m!} \Rightarrow$$
$$\sum_{m=0}^{\infty} P_m = f_2(t) \sum_{m=0}^{\infty} \frac{f_1(t)^m}{m!} = f_2(t)e^{f_1(t)}$$

So finally the probability for cells with zero proteins is given by:

$$P(p=0,t) = \sum_{m=0}^{\infty} P_m = \exp\left[\frac{\alpha}{\gamma_m+\beta}\left(\frac{\beta}{\gamma_m+\beta}\left(1-e^{-(\gamma_m+\beta)t}\right)-\beta t\right)\right] \tag{0.7}$$

After a short transient, this expression can be approximated by:

$$P(p=0,t) = \sum_{m=0}^{\infty} P_m = \exp\left[\frac{\alpha\beta}{(\gamma_m+\beta)^2}\right]\exp\left[\frac{-\alpha\beta t}{\gamma_m+\beta}\right] \tag{0.8}$$

For typical parameter values the coefficient $\exp\left(\frac{\alpha\beta}{(\gamma_m+\beta)^2}\right)$ is approximately 1 since $(\gamma_m+\beta)^2 > \beta\cdot\alpha$. So expression 0.8 is approximately given by:

$$P(p=0,t) = \exp\left(\frac{-\alpha\beta t}{\beta+\gamma_m}\right) \tag{0.9}$$

For high values of β this expression has a limit in $\exp(-\alpha t)$ which corresponds to the probability for getting a particular time interval or more given a Poisson process. This is simply the probability of not producing an mRNA, which is to be expected for a high protein production rate.
The correction to the mRNA Poisson process, given by $\frac{\beta}{\gamma_m+\beta}$ in equation 0.9, is the probability to produce a protein once the mRNA is produced instead of degrading that mRNA.

0.1.1 Time spend in the zero-protein state

In the previous paragraph, an approximate analytical expression for the population level probability for zero proteins as a function of time was derived. In the following, we extend this by calculating the single cell probability to be in that state for a specified time interval τ_c.
The fraction of cells with zero proteins at the start of the experiment can be given by the negative binomial approximation in equation 0.10 from [7].

$$P(n=0,t=0) = \left(\frac{1}{1+\beta/\gamma_m}\right)^{\alpha/\gamma_p} \tag{0.10}$$

The exit from the zero-protein state can be assumed to be governed by equation 0.9. So the probability

of staying a certain time span τ_c or longer is given by equation 0.11.

$$P(n = 0, \Delta T \geq \tau_c) = \exp\left(\frac{-\alpha\beta\tau_c}{\gamma_m + \beta}\right) \tag{0.11}$$

Finally, the fraction of surviving cells is obtained by multiplying expression 0.10 and 0.11, given in equation 0.12

$$f_{survival} = P(n = 0, \Delta T \geq \tau_c) \cdot P(n = 0, t = 0) = \exp\left(\frac{-\alpha\beta\tau_c}{\gamma_m + \beta}\right) \cdot \left(\frac{1}{1 + \beta/\gamma_m}\right)^{\alpha/\gamma_p} \tag{0.12}$$

Equation 0.12 represents the simple mathematical model presented in the article.

1 The tail effect in the autoregulated system

The tail effect is also present in autoregulated systems. A small change in the binding of the protein to its own promoter can greatly increase or decrease the number of rare concentrations in the distribution. This is first shown by changing the mean with the dissociation constant $K_D = \frac{k_{off}}{k_{on}}$, which in this study is done by changing the off-rate of the protein binding to its own promoter. This corresponds to a change in the binding affinity of the protein to the DNA and could easily be modified by small mutations in either the promoter of the protein binding region or in the protein itself.

1.1 Master equation for the autoregulated system

We model the autoregulated system by a two-state system, with the promoter being either unbound ("off"), which means the promoter is on, or bound ("on"), which means the promoter is off. This is similar to the system in Shahrezaei et al. [7]. The protein itself binds to and unbinds from the promoter at constant rates, given by k_{on} and k_{off}. Since the probability of binding is constant per protein, the actual on-rate is given by $n \cdot k_{on}$. The off-rate is a constant, which means that only one gene copy is present. Such a system is described by the master equation 1.1.
When a protein bound to the promoter is diluted, the system changes from bound to unbound. This is represented by the term $d \cdot P^{+}_{m,n+1}$ referred to as *dilution of bound protein.*

$$
\begin{aligned}
d_t P^{off}_{m,n} = &\ \alpha(P^{off}_{m-1,n} - P^{off}_{m,n}) && \textit{mRNA production} \\
&+ \gamma_m([m+1]\, P^{off}_{m+1,n} - mP^{off}_{m,n}) && \textit{mRNA degradation} \\
&+ \beta m(P^{off}_{m,n-1} - P^{off}_{m,n}) && \textit{protein production} \\
&+ \gamma_p([n+1]\, P^{off}_{m,n+1} - nP^{off}_{m,n}) && \textit{protein degradation} \\
&- k_{on} \cdot n \cdot P^{off}_{m,n} + k_{off} \cdot P^{on}_{m,n} && \textit{binding/unbinding} \\
&+ \gamma_p \cdot P^{on}_{m,n+1} && \textit{dilution of bound protein} \\
&\textit{for} \quad m \in [0, m_{max}] \quad n \in [0, n_{max}]
\end{aligned}
\tag{1.1}
$$

$$
\begin{aligned}
d_t P^{on}_{m,n} = &\ \gamma_m([m+1]\, P^{on}_{m+1,n} - mP^{on}_{m,n}) && \textit{mRNA degradation} \\
&+ \beta m(P^{on}_{m,n-1} - P^{on}_{m,n}) && \textit{protein production} \\
&+ \gamma_p([n+1]\, P^{on}_{m,n+1} - nP^{on}_{m,n}) && \textit{protein degradation} \\
&+ k_{on} \cdot n \cdot P^{off}_{m,n} - k_{off} \cdot P^{on}_{m,n} && \textit{binding/unbinding} \\
&- \gamma_p \cdot P^{on}_{m,n+1} && \textit{dilution of bound protein} \\
&\textit{for} \quad m \in [0, m_{max}] \quad n \in [1, n_{max}]
\end{aligned}
$$

1.2 A change in the mean

Changing the mean by modifying the dissociation constant of the autoregulating protein gives a strong tail effect, as shown in figure S1. A difference of ±50 percent changes the probability for rare events with several orders of magnitude for both a high ($n_{high} = 480$) and a low ($n_{low} = 80$) threshold.
The values of the dissociation constant correspond to a somewhat weak regulation. The values presented in figure S1 are between 100 and 1000 molecules per cell, while the mean is between 165 and 324. The mean without regulation is 429. So even for a somewhat weak regulation, there is a major tail effect.
As can be observed in figure S1, the stronger the regulation, i.e. a lower dissociation constant, the narrower the distribution. The same notion was made by Friedman et al. [2], that stronger negative autoregulation gives less cell-to-cell variation in protein distributions. The problem is that a decrease in the mean in a constitutive system will also give a smaller variance, as seen for the variance in the negative binomial approximation $\sigma^2 = \mu(1 + b)$. So to compare the effect of autoregulation on tails, we chose to compare distributions with approximately the same mean. This is similar to the study by

Rosenfeld et al. [6], where systems with the same mean were compared.

1.3 Constant mean but different shape

Protein distributions with the same mean, but different dissociation constants, were obtained as described in section 1.5. The solutions result in PMFs that are higher at the mean and decays faster for values away from the mean when the dissociation constant is low. This has a big effect on the tails and drastically influences the probability for rare concentrations, especially for the zero-protein phenotype as seen in figure S2B. The parameters to keep the constant mean are within $\alpha \in [0.05; 19]$ and $K_D \in [0.1; 1000]$.
Interestingly, below a limit around $K_D \approx 1$ the variance starts increasing, as shown in the figure S3. This makes the probability for both the high concentrations and the zero-protein state increase, as seen in figure S2. This is probably due to burstiness in the mRNA production. The variance and the Fano factor for the mRNA distribution increases below $K_D \approx 1$ as seen in the figures S3BC.
The Fano factor approaches 1 as the dissociation constant gets weaker, which is to be expected for the unregulated promoter, which produces mRNA as a Poisson process [8].
For $K_D \geq 1$, the larger the K_D the larger the variance, which increases both the probability of extremely low and high protein numbers.
Interestingly, the tail of high concentrations is less affected by changes in the dissociation constant. In the constitutive system, the far high concentration tail was more affected by changes to the translation step, than to the transcription step. Since autoregulation happens at the level of transcription, this could be related to that effect.

1.4 Transient overshoot during adaptation

The negatively autoregulated systems exhibit some transient phenomena after activation. The preceding results have shown phenomena related to steady-state distributions, but an autoregulated system has a new kind of tail effect during the evolution of the system from the zero molecules initial condition. The time evolution of the mean, given by the master equation 1.1, is shown in figure S4A. Following the activation of the system, there is a significant overshoot in the mean protein level, before the steady state is reached. This is because there is some delay from the first production of mRNAs to the production of a protein, which means that too many mRNAs, relative to the mean level, can be produced before the system autoregulates.
As described in section 1.5 pairs of the dissociation constant K_D and the mRNA production rate α are chosen such that the steady-state mean is kept constant at 50 proteins per cell. The values are within $\alpha \in [0.5; 19]$ and $K_D \in [0.1; 5.0]$. The maximum overshoot appears in the system with a very high mRNA production rate and very low dissociation constant, which means a strong autoregulation.

But the overshoot is only 2-fold, with the mean level transiently exceeding 100 proteins, as shown in figure S4A. On the other hand, if we choose a rare phenotype of $n > \tau = 450$ and look at the probability as a function of time, given by $Pr(t, n \geq \tau) = \sum_{n=\tau}^{\infty} P(t, n)$, the result is drastically amplified. As shown in figure S4B, the probability for the occurrence of a rare concentration increases with orders of magnitude. For the most strongly regulated system, the difference in the occurrence of rare concentrations is more than 3 orders of magnitude different at its maximum value, as shown in figure S4C.

1.5 How to obtain the same mean for various dissociation constants

Since no analytical solution exists to equation 1.1, it is not easy to obtain the same mean for each of these distributions. First, an array of dissociation constants were chosen. Now, the task is to determine a corresponding array of transcription rates, α, that give the same mean. The transcription rate was obtained from Friedman's approximation [2]:

$$P(x) = Ax^{a-1}e^{-x/b}\left(1 + x/K_D\right)^{-a}$$

First the expression was numerically integrated from zero to infinity to determine $A_{K_D,\alpha}$, i.e. the specific normalization constant associated with K_D and α given by $A_{K_D,\alpha}^{-1} = \int_0^{\infty} P(x \mid K_D, \alpha)dx$. Next the mean was determined using the integral: $\mu_{K_D,\alpha} = \int_0^{\infty} x \cdot P(x \mid K_D, \alpha)dx$.
Using these values for the exact numerical solution, gave somewhat imprecise results with a mean of 50 ± 2. To get more precision, a series of solutions to the appropriate master equation were obtained, with incremental changes in α. A linear fit to these values finally gave the target α value and yielded means of 50 ± 0.5.

2 The tail effect with soft thresholds

Probability distribution tails are in general more affected by small changes than the mean part of the distribution. As such, it is not surprising to observe this effect in protein distributions. However, the main task lies in how to relate the protein distribution tails to the formation of rare phenotypes. This requires a more careful consideration of what constitutes the threshold between a main and a rare phenotype.

The analysis in the main text relies on a hard threshold in the definition of a rare concentration, which means that the difference between a rare and a main concentration corresponds to a difference of one protein. For example, a cell with 10 proteins could be categorized as *rare*, while a cell with 11 proteins could fall in the category of the main population. This threshold between concentrations does not necessarily correspond to a threshold of phenotypes, in fact, it is likely too idealized.

However, we do find possible examples of relatively hard thresholds in biology, with candidates such as phage attack evasion by not containing the appropriate receptor, toxin-antitoxin systems, and translational regulation by small RNAs. The first example is described in the main text, whereas the last two are examples of regulation by heterocomplex formation, where a given substrate is regulated by the concentration of another substrate. In the case of toxin-antitoxin regulation, the function of the toxin is dependent on whether or not it is bound by an antitoxin. Once the level of toxin exceeds the level of antitoxins, the cell can enter a toxin-dominated state, which has been associated with transient tolerance to antibiotics [1]. The regulation by small RNAs can in some cases exhibit ultrasensitive regulation, once the level of sRNA exceeds the level of mRNA [4].

Depending on the system of interest, the threshold defining a rare phenotype might be a lot softer than the hard threshold described in the main text. Other possible softer thresholds might define a rare phenotype, such as a Hill function, an exponential function or a more coarse-grained log-scale threshold. These are investigated in the following sections, after the mathematical definition of a threshold is given.

Mathematical definition of thresholds

We define a threshold as a function that picks out a subset of the probability mass function. The probability for a cell to have a rare phenotype is then given in equation 2.1.

$$Pr(\tau) = \int_{-\infty}^{\infty} T(x \mid \tau) \cdot P(x) dx \tag{2.1}$$

Where the function $T(x \mid \tau)$ defines the threshold. The parameter τ defines the location or scale of the threshold between rare and regular phenotypes.

For a hard threshold the function $T(x \mid \tau)$ is simply a step-function defined by equation 2.2.

$$\text{High threshold: } T(x \mid \tau) = \begin{cases} 1 \text{ for } x \geq \tau \\ 0 \text{ for } x < \tau \end{cases} \tag{2.2}$$

$$\text{Low threshold: } T(x \mid \tau) = \begin{cases} 0 \text{ for } x > \tau \\ 1 \text{ for } x \leq \tau \end{cases}$$

Hill function threshold

A cooperative system of regulating proteins can contain a sharp transition from a state where most proteins are unbound to a state where most proteins are bound as oligomers. This is typically modelled by a Hill function as given in equation 2.3, where the transition becomes sharper the higher the Hill coefficient H.

$$\text{High threshold: } T(x \mid \tau) = \frac{x^H}{\tau^H + x^H} \tag{2.3}$$

$$\text{Low threshold: } T(x \mid \tau) = \frac{\tau^H}{\tau^H + x^H}$$

As seen in figure S5, for $H = 8$, this type of threshold do not lead to a big tail effect. A hill coefficient of 8 corresponds to eight proteins in the regulating complex, which can be considered a very high number and thus should lead to a sharp transition. A change of fifty percent in the protein level, leads to less than two orders of magnitude of probability. As such, a threshold defined by a Hill function do not lead to ultrasensitivity.

Exponential function threshold

An extension of the phage evasion example, where the presence of a receptor leads to phage infection, could be a softening of the hard threshold, by assigning a fixed probability to be infected per receptor. Given a fixed probability to infect per receptor $1 - p_0$, the probability to survive would be

the probability p_0 to the power of the number of receptors(n): $P_{survival} = p_0^n = exp(n \cdot log(p_0)) = exp(-n \cdot log(1/p_0))$. Depending on the probability of survival per receptor, this threshold would be more or less sharp. The lower the probability, the harder the threshold. This is shown in figure S6. The threshold is given by the equations

$$\text{High threshold: } T(x \mid \tau, \lambda) = min\left[exp(\lambda \cdot (x - \tau), 1\right] \tag{2.4}$$

$$\text{Low threshold: } T(x \mid \tau, \lambda) = \begin{cases} exp(-\lambda \cdot (x - \tau)) \text{ for } x \geq \tau \\ 1 \text{ for } x < \tau \end{cases} \tag{2.5}$$

Log-scale threshold

The defining threshold for rare phenotypes could be more coarse-grained than the difference between plus minus a single protein. The threshold could be on a log-scale, where the importance was in the difference between e.g. a hundred and a thousand proteins. In such a case the tail effect is no longer present. For example, the probability for a cell to have zero proteins in an unregulated system was modelled by $P(0) = \left(\frac{1}{1+b}\right)^a$, which on a log-scale is only linearly dependent on the parameter a.

Bibliography

[1] Ilaria Cataudella et al. "Conditional Cooperativity of Toxin - Antitoxin Regulation Can Mediate Bistability between Growth and Dormancy". In: 9.8 (2013). DOI: 10.1371/journal.pcbi.1003174.

[2] Nir Friedman, Long Cai, and X. Sunney Xie. "Linking stochastic dynamics to population distribution: An analytical framework of gene expression". In: *Physical Review Letters* 97.16 (2006), pp. 1–4. ISSN: 00319007. DOI: 10.1103/PhysRevLett.97.168302.

[3] N.G. Van Kampen. *Stochastic Processes in Physics and Chemistry*. 3rd editio. Elsevier Science & Technology Books, 2007. ISBN: 0444529659.

[4] Namiko Mitarai et al. "Dynamic features of gene expression control by small regulatory RNAs." In: *Proceedings of the National Academy of Sciences of the United States of America* 106.26 (2009), pp. 10655–9. ISSN: 1091-6490. DOI: 10.1073/pnas.0901466106. arXiv: arXiv:1408.1149.

[5] K. F. Riley and M. P. Hobson. *Essential Mathematical Methods for the Physical Sciences*. Cambridge University Press, 2011, pp. 228–272.

[6] Nitzan Rosenfeld, Michael B. Elowitz, and Uri Alon. "Negative autoregulation speeds the response times of transcription networks". In: *Journal of Molecular Biology* 323.5 (2002), pp. 785–793. ISSN: 00222836. DOI: 10.1016/S0022-2836(02)00994-4. arXiv: 0412010 [q-bio].

[7] Vahid Shahrezaei and Peter S. Swain. "Analytical distributions for stochastic gene expression". In: 2008 (2008). ISSN: 0027-8424. DOI: 10.1073/pnas.0803850105. arXiv: 0812.3344.

[8] Yuichi Taniguchi et al. "Sensitivity in Single Cells". In: *Science (New York, N.Y.)* 329.5991 (2011), pp. 533–539. ISSN: 1095-9203. DOI: 10.1126/science.1188308.
