# Supplementary figures and images for: Gene Expression Changes with Minor Effects on the Population Average Have Major Effects on the Occurrence of Cells with Extreme Protein Concentrations
Source: mSphere. 2019 Jan 30;4(1):e00575-18. doi: 10.1128/mSphere.00575-18 (PMC6354807; doi:10.1128/mSphere.00575-18)

$K_D = 50$ 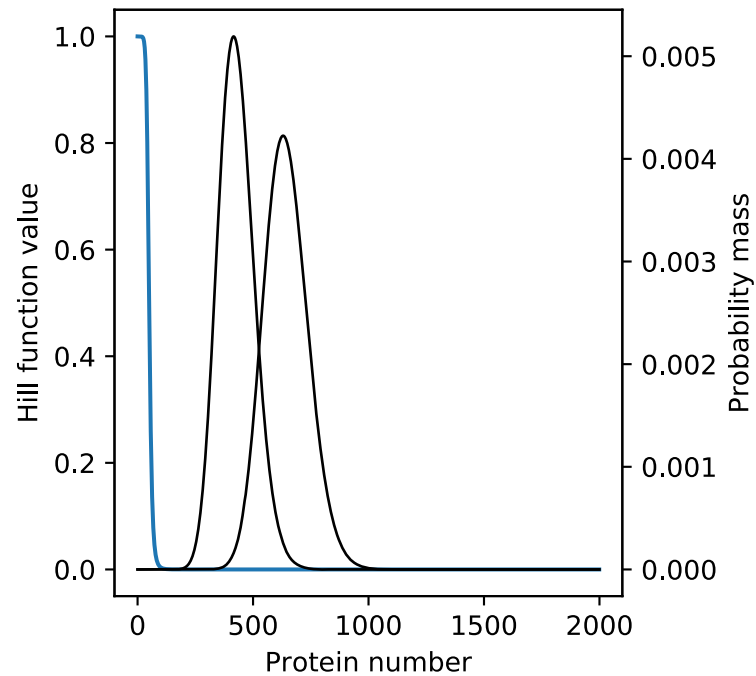 $K_D = 5000$ 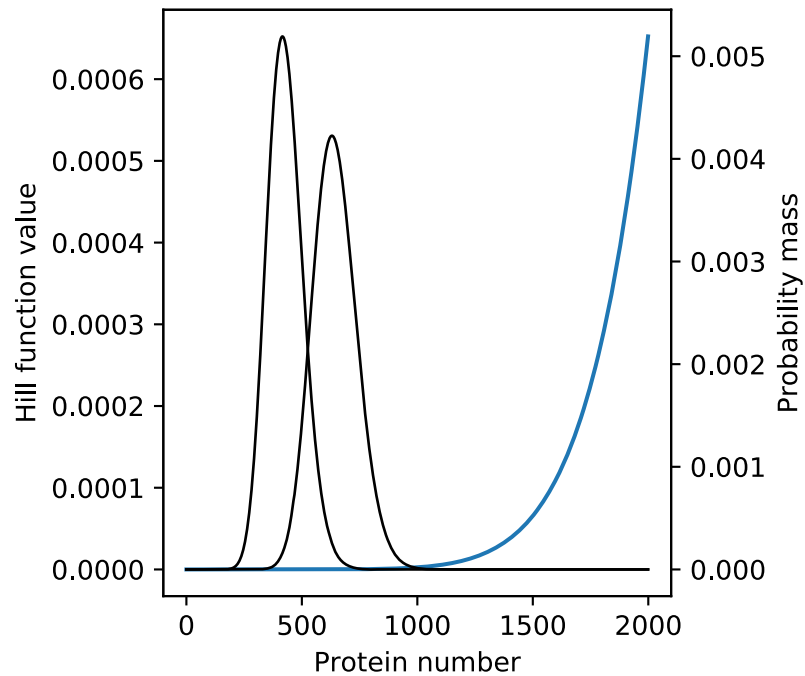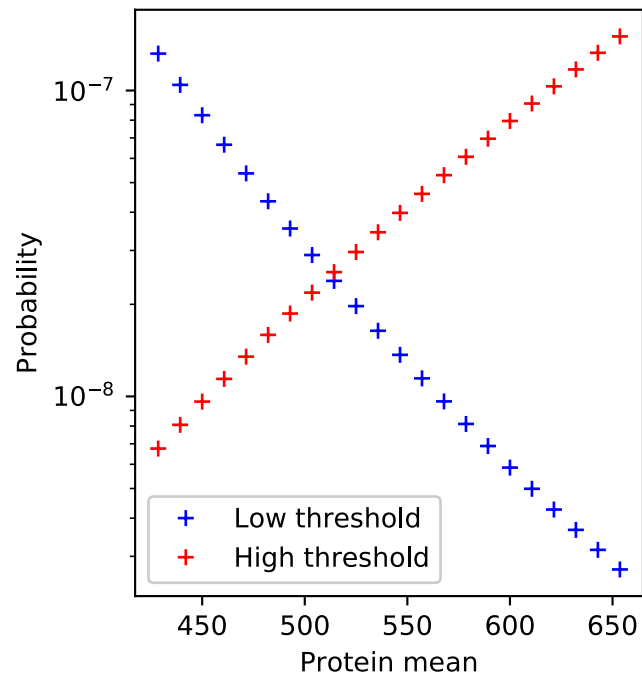

Supplement: FIG S5 [file mSphere.00575-18-sf005.pdf]
